# Social media use, online racism, and mental health among Black Emerging Adults in the United States

**DOI:** 10.1371/journal.pdig.0001243

**Published:** 2026-08-13

**Authors:** Oreoluwa O. Badejoh, Shedrick L. Garrett, Melissa C. Holland, Vanessa V. Volpe, Brian TaeHyuk Keum, Jimi Huh, Hans Oh

**Affiliations:** 1 . Department of Psychology, North Carolina State University, Raleigh, North Carolina, United States of America; 2 . Department of Psychology, University of North Carolina at Chapel Hill, Chapel Hill, North Carolina, United States of America; 3 . School of Public Health, University of California at Berkeley, Berkeley, California, United States of America; 4 . Keck School of Medicine, University of Southern California, Los Angeles, California, United States of America; 5 . Suzanne Dworak-Peck School of Social Work, University of Southern California, Los Angeles, California, United States of America; Wollo University, ETHIOPIA

## Abstract

Social media use is a key risk factor for mental health symptomatology among emerging adults. Black emerging adults use social media frequently, where they are exposed to online racism, which may contribute to worse mental health. The goal of the current study was to examine the frequency of exposure to online racism as a mediator in the association between the frequency of social media use and depression and anxiety symptoms. Using a non-probability sample of 1005 monoracial Black emerging adults (*M*_age_ = 24.07, 50.6% women) from a larger investigation, participants completed an online survey, in which they reported their social media use, exposure to online racism, and mental health symptoms using established measures. Findings support mediation of associations for some social media platforms. More frequent Twitter (standardized indirect effect (SIE):.04, *p* = .027), Reddit (SIE:.10, *p* < .001), and TikTok (SIE:.05-.06, *p* = .005) use were associated with more frequent exposure to online racism, and more frequent exposure to online racism was associated with increased odds of depression and anxiety. Mediation was not supported for YouTube, Facebook, or Instagram. Clinicians should consider stress from online racism as a factor for clients with high use of certain social media platforms. Future research can explore whether platform features like content warnings, hate speech moderation, and bias response teams could also help reduce exposure.

## Author summary

Black emerging adults are among the most active social media users, making these platforms an important space for identity development, social connection, and community. However, these online environments can also expose users to racism that may negatively affect mental health. In this study, we examined whether exposure to online racism helps explain the relationship between social media use and symptoms of depression and anxiety among Black emerging adults. We found that more frequent use of Twitter/X, Reddit, and TikTok was associated with greater exposure to online racism, which in turn was linked to higher levels of depression and anxiety. In contrast, this pattern was not observed for Instagram, Facebook, or YouTube, suggesting that the mental health impact of social media differs across platforms. These differences may reflect platform-specific features such as content moderation policies, recommendation algorithms, anonymity, and network structures that influence users’ exposure to racist content. Our findings highlight the need for clinicians, researchers, policymakers, and technology companies to consider how platform design and moderation practices can reduce exposure to online racism and better protect the mental health of Black emerging adults.

## Introduction

In the United States, depression and anxiety continue to be critical concerns for emerging adult populations. Emerging adulthood (ages 18–29) is a distinct developmental period characterized by increased independence, decreased parental support, and the ushering in of adult responsibilities and expectations that can strain mental health [1, 2]. For instance, the 12-month prevalence rate for psychiatric disorders is highest between ages 18 and 29 years [3]. Coupled with the developmental transitions of emerging adulthood, Black emerging adults also face exposure to race-related stressors (e.g., discrimination, stereotype threat) that pose increased risk for depression and anxiety [4]. Depression and anxiety symptoms are common for Black emerging adults, with one investigation of Black college-attending emerging adults reporting that as many as 83% to 90.2% have at least one depression symptom and 60.7% to 71.5% have at least one anxiety symptom [5]. There have also been significant increases in the prevalence of depression and anxiety since 2008 [6, 7]. Therefore, understanding correlates of depression and anxiety symptoms and mechanisms through which these correlates may confer mental health risk for Black emerging adults remains an important priority that can inform intervention efforts and clinical practice.

One potential correlate of depression and anxiety symptoms is social media use. Social media, defined as forms of digital communication through which users create online communities to share information, ideas, personal messages, and more, is a key developmental context for emerging adults [8, 9]. Eighty four percent of Black emerging adults report using social media, with over half being active on social media platforms several times a day [10], often for at least three hours [11]. Among the most popular social media platforms, most Black adults report using YouTube (88%), Facebook (73%), Instagram (52%), and TikTok (50%) [12]. Twitter (currently known as “X”) and Reddit also maintain moderate levels of popularity among Black emerging adults, with 24% and 18% of Black adults reporting some use of these platforms [10; 12]. Research has indicated a link between social media use and mental health, such that more frequent social media use is associated with worsening mental health among emerging adults [13, 14], while some find no association between social media use and mental health symptoms [15, 16]. Additionally, investigating the types of experiences had on social media beyond amount of social media use- including exposure to content and social media interactions - may shed light on when social media use has positive versus negative associations with mental health. Therefore, in the current study, we aim to understand if negative experiences on social media (i.e., online racism) are one potential explanation for the association between more frequent social media use and worse mental health.

**Fig 1:**
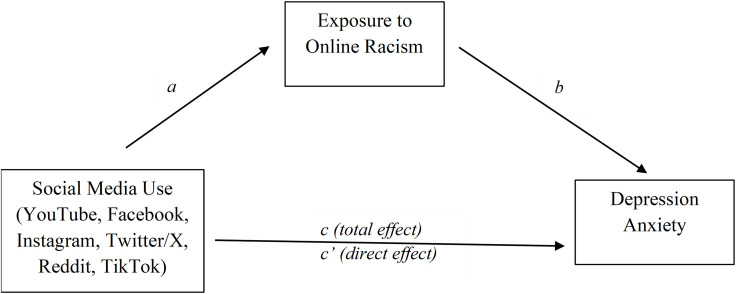
Conceptual mediation model of associations between frequency of social media use by platform and depression or anxiety.

For Black emerging adults, social media use can lead to online racism, which may explain part of the association between more frequent social media use and more depression and anxiety. Although members of other racial/ethnic groups also experience online racism, in the current study, we focus on the experiences of Black emerging adults as one heterogeneous marginalized group with high mental health risk and high rates of social media use. Exposure to anti-Black racism (hereafter “racism”) is defined as experiencing or witnessing differential and negative treatment of and cultural messages about Black individuals [17]. Racism can manifest within social media, where engagement in racism may be more overt and anonymous than in offline settings, and the algorithmic nature of social media platforms makes content rapidly shareable and widespread [18, 19]. Exposure to online racism can include exposure to negative, hateful, and threatening content about Black people and racially discriminatory interactions online, which can be experienced directly (e.g., be on the receiving end of a negative comment) or indirectly (e.g., viewing negative comments about another Black individual) [20]. These exposures are common, with previous studies finding that most Black emerging adults report experiencing online racism at least several times per year [21, 22] and traumatic online content many days of the week [23]. Frequent exposure to online racism has been associated with worse mental health for Black emerging adults, including more symptoms of depression and anxiety [24–26]. However, research on online racism as a potential explanation for the association between more frequent social media use and depression and anxiety among Black emerging adults is scarce. We aimed to fill this gap in two ways: (1) by examining online racism as a mediator (Fig 1), and (2) by examining platform-specific social media use, rather than grouping all social media platforms together into a general measure of social media use. Discerning between social media platforms may provide specific implications for social media policy and intervention efforts.

### Social media use and mental health

Bodies of literature specific to those individuals who use social media more frequently than is typical for the general population, termed problematic social media use [e.g., 27–28] or internet addiction [29], find negative mental health consequences of more use. For instance, problematic social media use is associated with greater depressive symptoms among a nationally-representative sample of emerging adults [30]. However, these associations may be specific to the context of excessive use, which can include compulsive or dependent use behavior that engenders withdrawal and loss of control [29].

Much of the literature on social media use and poor mental health to date has been conducted with majority-White samples, necessitating additional research on explanations for associations between social media use and mental health among the Black emerging adult population. The small body of existing research on this subject has found that the frequency of Black emerging adults’ social media use impairs well-being. In unpublished investigations, excessive weekly use of social media was associated with more distress among Black emerging adults [31], and more frequent use of Instagram among Black emerging adult women was associated with more body image dissatisfaction [32]. More frequent Instagram use is associated with higher levels of distress due to hurt feelings among Black emerging adults [33]. In terms of mental health symptoms, Black emerging adults who report high levels of Facebook use are more likely to exhibit social interaction anxiety [34], aligned with results indicating that higher social media use is associated with higher symptoms of dispositional anxiety in a general emerging adult sample [35]. Rather than social media itself, aspects of the experience of Black emerging adult users while on social media may explain these findings, highlighting the benefits and drawbacks of social media for Black emerging adults.

### Associations between social media use and mental health through exposure to online racism

More frequent social media use may be associated with worse mental health because being online more frequently exposes Black emerging adults to the stressor of online racism. According to racial trauma theory [36], exposure to racism – whether offline or online – exerts a psychological toll on the individual who is directly or indirectly the target. Black emerging adults’ online experiences are often marked by exposure to racist discussions and content [37]. A majority of studies describing the experience of Black social media users and mental health focus on the negative mental health consequences of exposure to online racism, trauma, and victimization [25, 38–45]. Social media use may disrupt mental health when social media experiences are racially discriminatory, traumatic, or stressful, as these experiences engender negative emotions, rumination, and threaten self-worth [46]. More frequent social media use may also decrement mental health if it increases exposure to traumatic or discriminatory content about Black people that activates anticipatory race-related psychological and physiological stress responses [37], which have been associated with worse mental health outcomes in offline settings [e.g., 47, 48]. In one previous investigation of racially/ethnically marginalized emerging adults, exposure to online racism mediated the association between problematic internet use and symptoms of depression and anxiety [28]. Those with greater problematic internet use had greater exposure to online racism, which was in turn associated with more symptoms of depression and anxiety [28]. This paper sets to explore these associations with general social media use rather than problematic use.

First, more frequent social media use may be associated with more exposure to online racism (Fig 1, path “a”), but the degree to which associations are platform-specific remains understudied. Research has found that Black emerging adults on Twitter and Facebook who report more time on social media have more frequent exposure to online racism [43, 37]. Other research suggests that Black emerging adults with higher average social media use across platforms such as Facebook, Twitter, Instagram, and Snapchat also report more frequent exposure to online racism [21]. However, the degree to which (1) more frequent use of other social media platforms is associated with more frequent exposure to online racism, and (2) more frequent use of a given social media platform after accounting for use of another is associated with more frequent exposure to online racism, requires further examination.

Next, more frequent exposure to online racism may be associated with depression and anxiety (Fig 1, path “b’). Black emerging adults who have experienced online racism report greater symptoms of psychological distress, depression, and social anxiety [39,24]. More frequent online racism on particular platforms, including Twitter (now known as X), Instagram, YouTube, TikTok, and Facebook, has also been associated with greater depressive and anxiety symptoms among Black emerging adults [49], which differences may be attributed to specific content moderation policies related to detecting hate speech [50]. As exposure to online racism has been linked to detrimental mental health for Black emerging adults, an understanding of this negative social media experience as an explanation for links between greater social media use and worse mental health can have implications for the development of clinical and technology-based interventions tailored for this population.

### Current study

The central aim of the current investigation is to understand the role of exposure to online racism in the association between social media use and mental health for Black emerging adults. Drawing from the racial trauma framework [36], we examined if online racism mediates the association between social media use and depression and anxiety (Fig 1).

Our hypothesis is as follows: There will be a significant indirect effect of online racism exposure, such that more frequent social media use will be associated with more frequent exposure to online racism (i.e., path “a”) and that more frequent exposure to online racism will be associated with greater odds of depression and anxiety (i.e., path “b”).

We also considered if there were significant associations between social media use and mental health variables for Black emerging adults (direct effect or total effect: “c” paths). Although we did not have distinct hypotheses for each social media platform, we expanded on the current literature by considering social media use separately for each social media platform (i.e., YouTube, Instagram, Facebook, Twitter, Reddit, TikTok) to better inform clinical and policy efforts.

## Materials and methods

### Ethics statement

This study was approved by the Institutional Review Board at the University of Southern California (IRB #APP-24–05114). All participants were anonymous and selected ‘agree’ to consent to participate in the survey.

Data come from a larger investigation of Black and Asian emerging adults (ages 18–29) residing in the United States, which was conducted online from July to September 2024. Participants were recruited via Qualtrics Panels, a participant panel aggregating research service, which generated a non-probability sample of individuals who met the inclusion criteria. Qualtrics Panels drew from Torfac, PureSpectrum, and Lucid participant panels and employed quotas to ensure adequate representation of gender, socioeconomic status, and geographic region of Black and Asian emerging adult participants in the sample.

Eligible participants completed an online survey. Data quality was ensured in several ways. First, each panel confirmed participants’ credentials (i.e., respondent address, demographic information, and email address) using third-party validation platforms (e.g., TrueSample, RelevantID, USPS) to ensure verified participants were enlisted. Second, Qualtrics Panels also removed those who completed the survey too quickly, straight-line responders, and multiple respondents from the same IP address. Third, a reCAPTCHA bot check was used to screen out potential non-human responses. Fourth, two attention check questions were embedded into the survey—participants were asked to verify which item was a vegetable in a list of food items and select ‘strongly agree’ on a Likert scale to show they were paying attention. Participants who did not meet data quality checks were excluded. Participants who met the checks and completed the survey were compensated directly by the participant paneling service. Compensation options included rewards such as cash, airline miles, gift cards, redeemable points, charitable donations, sweepstakes entrance, and vouchers.

### Participants

To answer our research questions, we utilized the subsample of 1005 monoracial Black emerging adult participants (*M*_*age*_ = 24.07; *SD*_*age*_ = 3.04) from the larger study. The proportion of women (50.6%) and men (49.1%) were approximately equal, and there were a small number of participants who identified as transgender, nonbinary, or genderfluid (.3%) in this analytic sample. Most participants had regular Internet access at work or home (98.3%), and 60.7% reported using the Internet almost constantly during the day.

## Measures

### Depression and anxiety

Depression and anxiety were each measured using two self-report scales that were used to index clinically significant mental health challenges. The Patient Health Questionnaire-9 (PHQ-9; [51]) was used to measure the degree to which participants were bothered by nine common depressive symptoms (e.g., “little interest or pleasure in doing things,”) over the past two weeks on a Likert-type scale from 1 (*Not at all*) to 4 (*Nearly every day*). The General Anxiety Disorder 7-item scale (GAD-7; [52]) was used to measure the degree to which participants were bothered by seven common anxiety symptoms (e.g., “feeling nervous, anxious, or on edge”) over the past two weeks on a Likert-type scale from 1 (*Not at all*) to 4 (*Nearly every day*). As we were primarily concerned with clinically-significant depression (sample 𝛼 = .90) and anxiety (sample 𝛼 = .92) in the current study, each continuous sum scale score was dichotomized to correspond to established clinical cutoffs for major depressive disorder (PHQ-9 score >= 10) and general anxiety disorder (GAD-7 score >=10), respectively. In sensitivity analyses, we also considered continuous PHQ-9 depression and GAD-7 anxiety symptom scores. When using continuous scores as outcomes, results were not substantively different. Depression or anxiety scores exceeding the cutoffs were coded as 1, and scores below the cutoffs were coded as 0. Both measures have been found to be valid for diverse populations, including Black adults [53, 54].

### Exposure to online racism

We used the Perceived Online Racism Scale - Very Brief [55] to index exposure to online racism. This six-item scale asks participants to report the frequency with which they experience online racism on a Likert-type scale from 1 (*Never*) to 5 (*Always*). Example items include “see online videos that portray my racial/ethnic group negatively” and “receive posts with racist comments.” According to scale specifications, we calculated a mean score (sample 𝛼 = .90). A higher score represents more frequent exposure to online racism.

### Social media use

The frequency of using specific social media platforms was assessed using six items developed for the survey. Participants were asked how often they use YouTube, Instagram, Facebook, Twitter (X), Reddit, and TikTok on a typical day, rating the frequency of use of each platform using a Likert-type response scale from 0 (*Not at all*) to 4 (*Almost constantly*). These platforms were considered because they were among the most popular platforms used by Black emerging adults when data were collected [12]. We considered each item as a separate predictor variable in our model to examine platform-specific associations, controlling for the use of other platforms. Higher scores indicate more frequent daily use of that social media platform.

### Sociodemographic covariates

We included sociodemographic covariates of age, gender, and health insurance status in our analytic models. We also did consider education level and employment status as potential sociodemographic covariates. However, when we tested them in the current sample, they were not statistically significantly associated with depression (education 𝜒2(7) = 5.32, *p* = .621.; employment 𝜒2(1) =.10, *p* = .755) or anxiety (education 𝜒2(7) = 5.66, *p* = .580; employment 𝜒2(1) =.38, *p* = .539). Therefore, we did not retain them in our analytic models. Some studies suggest that younger Black emerging adults face particular mental health risks compared to older Black emerging adults [56]. Therefore, in the current study, participants were asked to indicate their age in years. Gender differences in mental health among Black adults have been noted [57]. Therefore, participants selected their gender from a list of options, including “woman,” “man,” and an option to write in something else. Write-in responses were grouped and recoded such that a final third gender category was “transgender/nonbinary/fluid.” Health insurance is associated with socioeconomic status [58] and thereby contributes to mental health symptoms and treatment for emerging adults [59]. Participants self-reported if they currently had any form of healthcare coverage or health insurance (0 = no, 1 = yes, including public, private, or both).

### Analytic plan

We conducted a post-hoc Monte Carlo power analysis using the most conservative observed effect sizes (i.e., smallest correlations between predictors, mediators, and outcomes) and standard deviations, 10000 replications, 20000 Monte Carlo draws per replication, a confidence level of 95%, and a sample size of 1005. Our power was 89% to detect the most conservative indirect effect.

There was a small amount of missing data. Eight participants (.8% of the analytic sample) had missing data on gender, but there were no missing data on any other variables. Participants with missing data were not significantly different from those without data on sociodemographics, exposure to online racism, or social media use variables. Therefore, we deemed data missing at random and used maximum likelihood estimation to handle missing data in our analyses.

We first described the social media use, exposure to online racism, and depression and anxiety of the sample using descriptive statistics. Next, to answer our central research questions, we examined mediation using binary logistic regression path analysis models run in Mplus. As having depression and anxiety were strongly associated in the current sample (φ = .609, *p* < .001), we modeled each outcome in a separate model. Both models utilized maximum likelihood estimation, were run with 10,000 bootstrapped samples, and controlled for age, gender, and health insurance. Mediation was assessed using model indirect commands, which generates estimates of separate indirect effects as the product between each predictor and the mediator. Bias-corrected bootstrap confidence intervals were generated around this indirect effect, which remains an optimal approach in our relatively large analytic sample and given the default use of non-informative priors in Mplus [60, 61]. Of note, mediation analysis with cross-sectional data is justifiable when there is a strong guiding theory, statistical assumptions are met, and appropriate cautions about causality are used to contextualize the findings [62, 63]. The current study meets each of these criteria. Therefore, in the current investigation, we examine mediation as a first step to advance empirical support for subsequent longitudinal research.

## Results

### Descriptive statistics

On average, participants reported using YouTube many times a day (*M* = 3.12), Instagram (*M* = 2.67), and TikTok (*M* = 2.48) between a few and many times a day, Facebook (*M* = 2.13) and Twitter/X (*M* = 1.92) a few times a day, and Reddit less than a few times a day (*M* = 1.28). They reported exposure to online racism between “sometimes” and “half the time” in the past six months (*M* = 2.58). About half of the sample had PHQ-9 scores indicating depression (49.5%), and about 40% had GAD-7 scores indicating anxiety (42.4%). See Table 1 for sample characteristics.

**Table 1:** Characteristics of Analytic Sample of Black Monoracial Emerging Adults (*N* = 1005).

	M (SD)	n (%)
Age	24.07 (3.04)	
Gender		
Woman		504 (50.6%)
Man		490 (49.1%)
Transgender/Nonbinary/Fluid		3 (.3%)
Internet Use		
Not at all		9 (.9%)
< A few times a day		25 (2.5%)
A few times a day		107 (10.6%)
Many times a day		254 (25.3%)
Almost constantly		610 (60.7%)
Region		
Midwest		203 (20.2%)
Northeast		235 (23.4%)
South		481 (47.9%)
West		86 (8.6%)
Health insurance		
No		111 (11%)
Yes		894 (89%)
Internet access at home or work		
No		17 (1.7%)
Yes		988 (98.3%)
Social media use		
YouTube	3.12 (1.04)	
Instagram	2.67 (1.33)	
Facebook	2.13 (1.55)	
Twitter (X)	1.92 (1.53)	
Reddit	1.28 (1.40)	
TikTok	2.48 (1.48)	
Exposure to online racism	2.58 (1.10)	
Depression		
No		508 (50.5%)
Yes		497 (49.5%)
Anxiety		
No		579 (57.6%)
Yes		426 (42.4%)

***Note:*** Social media use responses were made on a Likert-type scale with the following options: 0 (Not at all), 1 (Less than a few times a day), 2 (A few times a day), 3 (Many times a day), and 4 (Almost constantly).

### Mediation

Table 2 and Table 3 reports the direct, indirect, and total effects of the mediation models. Covariates, social media use, and exposure to online racism together accounted for 18.3% of the variance in depression and 14.9% of the variance in anxiety.

**Table 2:** Direct Effects of Social Media Use on Depression and Anxiety through Exposure to Online Racism (N = 1005).

		Depression		Anxiety		
Direct Effects	OR^a^ or β	95% CI	p	OR or β	95% CI^b^	p
**Outcome:** **Mental Health Variable**						
YouTube Use	1.02	[.89, 1.16,]	.823	1.04	[.82, 1.26]	.600
Instagram Use	.81	[.72,.92]***	<.001	.94	[.84, 1.06]	.266
Facebook Use	1.10	[.99, 1.22]	.085	1.04	[.94, 1.15]	.484
Twitter Use	.97	[.87, 1.14]	.619	1.03	[.92, 1.16]	.578
Reddit Use	1.18	[1.06, 1.32]**	.007	1.05	[.94, 1.17]	.433
TikTok Use	.98	[.87, 1.08]	.652	.98	[.88, 1.09]	.693
Online Racism	1.98	[1.74, 2.31]***	<.001	1.90	[1.46, 2.48]***	<.001
Age	.96	[.92, 1.01]	.098	.97	[.93, 1.02]	.200
Gender	1.19	[.88, 1.58]	.285	1.27	[.94, 1.67]	.151
Insurance	.95	[.60, 1.51]	.820	1.13	[.73, 1.81]	.607
**Outcome:** **Online Racism**						
YouTube Use	-.01	[-.07,.05]	.665	-.01	[-.07,.05]	.666
Instagram Use	.01	[-.06,.08]	.705	.01	[-.06,.08]	.705
Facebook Use	.00	[-.07,.07]	.944	.00	[-.07,.07]	.945
Twitter Use	.09	[.01,.16]*	.020	.09	[.01,.16]*	.020
Reddit Use	.19	[.12,.26]***	<.001	.19	[.12,.26]***	<.001
TikTok Use	.11	[.04,.18]**	.002	.11	[.04,.18]**	.002
Age	-.02	[-.08,.04]	.522	-.02	[-.08,.04]	.522
Gender	.06	[-.01,.12]	.091	.06	[-.01,.12]	.089
Insurance	.03	[-.03,.09]	.341	.03	[-.03,.09]	.340

*p < .05, **p < .01, ***p < .001; 95% CIs for direct and total effects are bootstrapped bias-corrected CIs around unstandardized estimates

^a^OR = Odds Ratio

^b^CI = Confidence Interval

**Table 3:** Indirect, and Total Effects of Social Media Use on Depression and Anxiety through Exposure to Online Racism (N = 1005).

Indirect Effects	UnStd^e^	Std^f^	95% CI^a^
YouTube->On R^b^- > Dep^c^	-.01	-.01	[-.05,.04]
Instagram->On R- > Dep	.01	.01	[-.03,.05]
Facebook->On R- > Dep	.00	.00	[-.03,.04]
Twitter->On R- > Dep	.04*	.03	[.01,.09]
Reddit->On R- > Dep	.10***	.07	[.06,.15]
TikTok->On R- > Dep	.06**	.04	[.02,.10]
YouTube->On R- > Anx^d^	-.01	-.01	[-.05,.03]
Instagram->On R- > Anx	.01	.01	[-.03,.04]
Facebook->On R- > Anx	.00	.00	[-.03,.03]
Twitter->On R- > Anx	.04*	.03	[.01,.08]
Reddit->On R- > Anx	.10***	.07	[.06,.14]
TikTok->On R- > Anx	.05**	.04	[.02,.09]
**Total Effects**	**UnStd**	**Std**	**95% CI**
YouTube Use - > Dep	.01	.00	[-.13,.15]
Instagram Use - > Dep	-.20**	-.13	[-.33,.08]**
Facebook Use - > Dep	.10	.07	[-.01,.20]
Twitter Use - > Dep	.02	.01	[-.10,.13]
Reddit Use - > Dep	.26**	.18	[.15,.39]
TikTok Use - > Dep	.03	.02	[-.08,.14]
YouTube Use - > Anx	.03	.02	[-.12,.18]
Instagram Use - > Anx	-.06	-.04	[-.19,.07]
Facebook Use - > Anx	.04	.03	[-.07,.14]
Twitter Use - > Anx	.07	.06	[-.04,.19]
Reddit Use - > Anx	.14*	.10	[.03,.26]
TikTok Use - > Anx	.03	.02	[-.08,.14]

95% CIs for direct and total effects are bootstrapped bias-corrected CIs around unstandardized estimates; *p < .05, **p < .01, ***p < .001.

^a^CI = Confidence Interval

^b^On R = Online Racism

^c^Dep = Depression

^d^Anx = Anxiety

^e^UnStd = unstandardized estimate

^f^Std = standardized estimate

The indirect effect of social media use on depression and anxiety through exposure to online racism was supported for use of Twitter (depression indirect effect = .04, *p* = .027, 95% CI [.01,.09]; anxiety indirect effect = .04, *p* = .027, 95% CI [.01,.08]), Reddit (depression indirect effect = .10, *p* < .001, 95% CI [.06,.15]; anxiety indirect effect = .10, *p* < .001, 95% CI [.06,.14]), and TikTok (depression indirect effect = .06, *p* = .005, 95% CI [.02,.10]; anxiety indirect effect = .05, *p* = .005, 95% CI [.02,.09]), but not YouTube, Instagram, or Facebook. More frequent daily use of Twitter (β = .09, *p* = .020, 95% CI [.01,.16]), Reddit (β = .19, *p* < .001, 95% CI [.12,.26]), and TikTok (β = .11, *p* = .002, 95% CI [.04,.18]) were each associated with more frequent exposure to online racism (i.e., *a* paths of the mediation model). In turn, more frequent exposure to online racism was associated with a 98% increased odds of depression (OR = 1.98, *p* < .001, 95% CI [1.74, 2.31]) and a 90% increased odds of anxiety (OR = 1.90, *p* < .001, 95% CI [1.46, 2.48]) (i.e., *b* paths of the mediation model).

Both the total effects (*c* paths) and the direct effects (*c’* paths) of Twitter and TikTok use on depression and anxiety were nonsignificant, providing support for full mediation. Similarly, the total but not the direct effect of Reddit use was significantly associated with anxiety, suggesting full mediation. However, the total effect and direct effect of Reddit use were each significantly associated with depression, suggesting partial mediation. More frequent daily Reddit use was associated with an 18% increased odds of depression (OR = 1.18, *p* = .007, 95% CI [1.06, 1.32]), above and beyond exposure to online racism. Finally, although online racism did not mediate the association between daily Instagram use and depression or anxiety, more frequent daily Instagram use itself was also associated with 19% decreased odds of depression (OR =.81, *p* < .001, 95% CI [.72,.92]).

## Discussion

Social media remains an important aspect of Black emerging adults’ online experiences. At an age where this population is undergoing various transitions, social media provides them a place to build a collective identity and belonging with others [64, 65]. Given their high rates of social media use [10], Black emerging adults may also be exposed to negative experiences on social media, such as online racism. This study makes a unique contribution to existing literature in two ways. First, it examines the role of online racism in the association between social media use and depression and anxiety, providing one potential explanation for the association between social media use and negative mental health outcomes. Second, our results inform how the use of specific social media platforms is differentially associated with mental health. Findings from our study highlight how online racism may be a stressor associated with worse mental health for Black emerging adults who frequent social media.

As hypothesized, use of certain social media platforms was associated with depression and anxiety through exposure to online racism. In a similar study with racial/ethnic minoritized emerging adults, Keum et al. [28] found that exposure to online racism explained the association between problematic internet use and depression and anxiety. We extend this research to consider the relative frequency of social media use by platform, rather than pathological (i.e., problematic use, addiction) social media use, extending support for findings to general populations of Black emerging adults beyond those who struggle with excessive internet use. In line with racial trauma, exposure to online racism is a potent chronic stressor that can decrease self-worth, increase rumination, and engender psychological distress [36]. In the same way, those who use specific social media platforms may be exposed to more frequent online racism, which then engenders racial trauma responses that ultimately increase the risk of having depression and anxiety in the long term.

Online racism explained the association between social media use and depression and anxiety for only three of the six social media platforms we examined: Twitter, Reddit, and TikTok. TikTok is a particularly unique social media platform compared to others, as algorithms within TikTok emphasize instantaneous content consumption through the curation of a personalized feed catered to one’s interests [66]. As one of the first platforms to emphasize audiovisual content tailored for users based on their preferences and interests [67], the algorithmic format can also expose Black users to negative race-related content, such as the widespread consumption of racist and white supremacist messaging [68, 69] that occurs more rapidly than on other social media platforms due to the short and widespread video format [68]. Additionally, the emphasis of audio meme templates compared to other social media platforms [70] can lead to the adoption of digital Blackface by non-Black users: the appropriation of Black bodies, sounds, and culture by non‐Black people online for humorous and playful purposes [71, 72]. Although digital Blackface itself is not unique to TikTok as a platform, non-Black users may specifically adopt and appropriate trending audios from Black creators, acting as a form of digital Blackvoice [73]. Indeed, in a study where a sample of Black emerging adults reported themes of online racism such as cultural appropriation and problematic viral trends, TikTok was the most referenced social media platform by participants on which these occur [71]. Such mockery of Black individuals through appropriation can engender negative feelings on self-esteem and mental health [74]. Additionally, online racism also explained the association between use of Reddit and Twitter on depression and anxiety. According to the transformation framework [75], social media platforms provide seven unique features that can explain peer communication and interaction [75]. One of these features–anonymity- is especially relevant to the platforms of Reddit and Twitter which gives users the freedom to identify as they wish (i.e., no “real-name” policies or suggestions) and more freely express ideals and feelings [76, 77], compared to platforms such as Instagram or Facebook. Anonymity online has been linked to increased rates of racism and hate speech as it allows individuals to post more freely [78; 18, 19]. As a result, as Black users frequent these platforms, exposure to excessive hate speech and language can elicit feelings of stress and anxiety [79].

On the other hand, online racism did not explain the association between use of Instagram or Facebook on depression and anxiety. Instagram and Facebook are more frequently used for social interactions and to connect with individuals that they know personally, such as family and friends, compared to Twitter, Reddit, and TikTok [80, 81]. Such tighter social media networks give users more control over the platforms they use by allowing connection with close friends and family and blocking or banning of users they do not want to associate with— compared to platforms such as X or Reddit, where this feature does **not** function in a similar manner. Instagram and Facebook allow users to make their accounts private to only specific audiences– preventing users from seeing potentially hateful content from individuals outside of close circles. Additionally, users can limit who can share content with them, preventing unwanted media from unknown individuals. Twitter, on the other hand, defaults content to be viewed publicly [82]. A study of Black emerging adult users found that, on average, they reported over 500 Facebook friends, with about 85% of these friends also being Black [83]. It is possible that Black users may use platforms like Facebook and Instagram to primarily connect with other Black individuals, which may serve to protect them against some exposure to online racism. Another explanation for how Facebook and Instagram could potentially shield Black users from racist content could be the adoption of stricter content moderation policies. Meta (i.e., the company that owns Facebook and Instagram), compared to other social media platforms such as Twitter, is more transparent about their detection of hate speech, and has released data sharing their increased ability to detect such speech over time [50]. Additionally, Facebook and Instagram have strict policies that remove content that violates their guidelines, helping to prevent and filter out exposure to online racism. In contrast, Twitter will often allow users to view hateful content with a warning put in place in advance and often will *only* remove its most severe content [50].

Online racism also did not explain the association between use of YouTube– the most reported platform by our sample– and depression and anxiety. YouTube has also been open in its removal of hate speech and racist content. Like Instagram and Facebook, it utilizes a strike system that informs users when they are in danger of violating guidelines and increases severity of punishment with higher violations [50]. As a result, Black emerging adults may be able to reap benefits of YouTube, such as a sense of belonging and connection with other Black individuals, communities, or affirming content, such as that from natural hair communities which helps to prevent exposure to online racism [84]. Overall, Facebook, Instagram, and YouTube emphasize proactive identification of potentially race-related hateful content rather than reliance on user reports of hateful content, compared to Twitter and Reddit [50] which could help deter exposure of such content to Black users.

Surprisingly, we also found that more frequent Instagram use was associated with lower chances of depression for Black emerging adults. It may be that certain features of Instagram specifically improve meaningful and positive social connections. For example, a previous systematic review of emerging adult users of Instagram and their mental health outcomes found that use of Instagram facilitated interactions that reduced loneliness by allowing users to share photos that elevated their self-acceptance and self-esteem, and as a result, helped curb the negative mental health effects of the platform [85]. On Instagram, young Black populations have been able to explore identity and belonging through the specific features of the platform such as Instagram stories and Instagram Carousel (i.e., slideshow-style content)– which have been adopted as way for Black users to engage in activism and shared ethnic identity expressions [86]. It may be that these features especially can elicit positive feelings about being Black or feelings of belongingness and connection online that support positive mental health.

### Limitations & future directions

Our study is not without limitations. First, a cross-sectional design was used to examine mediation. Although the position of variables in our model was informed by prior theory and this is a preliminary investigation, subsequent longitudinal or experimental investigations are needed to replicate the current findings, confirm directionality, and infer causation. Second, we used a validated and standardized self-report measure of online racism. Still, it does not allow us to differentiate the type of online racist content to which participants were exposed (e.g., witnessing content about police brutality versus directly receiving insults online). While the frequency of exposure to online racism is captured within this measure, there may be nuances in the different types of exposure, which may have different implications for mental health symptomatology (e.g., traumatic stress symptoms versus depressive symptoms). Third, Black emerging adults reported using several social media platforms (i.e., YouTube, Instagram, TikTok) many times a day. As our study used non-probability sampling, current participants may be more frequently online than Black emerging adults that are harder to reach; therefore, this digitally active sample may be more prone to encountering online racism in comparison to other Black emerging adults who may not be as frequently online. As passive versus active use of social media platforms can differentially impact mental health outcomes [87–89], future studies could further parse out how Black emerging adults interact with specific social media platforms. Additionally, our current sample consists of a within-group study of monoracial Black emerging adults. However, because there is heterogeneity within the Black community [90], we cannot generalize our findings to all Black emerging adult online users. Lastly, a majority of our sample consisted of cisgender individuals, leaving gaps in our understanding of intersectional experiences of online racism (e.g., online gendered racism) that may distinctly shape the online experiences of Black emerging adults. With these limitations in mind, subsequent research should investigate the role of intersectional factors (e.g., gender) in influencing both the online experiences of Black emerging adults but also how such varied experiences may affect their mental health. Finally, other investigations may consider these issues with other racial/ethnic groups to understand if results might differ or appear similarly in those groups.

## Conclusions and clinical implications

In summary, the current study considered the role of exposure to online racism as one underlying factor that could explain the relationship between social media use and mental health for Black emerging adults, a population that is frequently online. Findings underscore the importance of understanding the features or interactions unique to different platforms, namely Reddit, Twitter/X, and TikTok, that expose Black emerging adults to online racism and increase their risk of mental health challenges. This study contributes to practice, research, and policy in several ways. It may be useful for clinicians working with Black emerging adults to ask them to describe the role of their preferred social media platforms in their mental health to gain deeper insight as to who may be navigating particularly harmful platforms (i.e., Reddit, Twitter/X, TikTok) and for what reasons. In doing so, clinicians should be aware of platform differences and brainstorm platform-specific coping strategies (e.g., reviewing policies for reporting or blocking accounts, removing automatic notifications, prioritizing content created by trusted creators, blocking specific terms and hashtags) with clients that may benefit them if they experience online racism. This study also demonstrates that social media effects are not uniform and instead specific platforms, like Reddit, Twitter/X, and TikTok, may engender different relative risk of exposure to online racism. These results complement recent calls for improved precision for operationalizing social media behaviors, content, and experiences [91]. Researchers should survey use of and experiences on specific social media platforms to understand social media use across multiple social media platforms, as opposed to capturing a global measurement on social media behavior (i.e., screentime). Technology designers and policy makers can consider how specific features (e.g., comments section, content warnings) and policies (e.g., content moderation, bias response teams) of social media platforms may intentionally or unintentionally pose heightened risk for exposure to online racism, thereby monitoring, revising, and regulating their products to benefit public health. Parallel smaller-scale clinical work with individual Black emerging adult social media users and larger-scale technology policy protections are needed to reduce the impact of exposure to online racism and improve the mental health of this digitally-engaged population.
