# Supplementary material for: Social media use, online racism, and mental health among Black Emerging Adults in the United States
Source: PLOS Digit Health. 2026 Aug 13;5(8):e0001243. doi: 10.1371/journal.pdig.0001243 (PMC13472414; doi:10.1371/journal.pdig.0001243)
Supplement: Supplementary file 1 [file pdig.0001243.s002.docx]

Response to Reviewers

Thank you for the opportunity to submit a revision of our manuscript, entitled “Social media use, online racism, and mental health among Black Emerging Adults,” for consideration for publication in *PLoS Digital Health.*

We thank the editors and reviewers for recognizing the importance of this topic and for their valuable feedback, which we believe has substantially contributed to this work. We have created an itemized list of each comment and how it was addressed below. Comments are organized by the reviewer’s response to each review question.

**Editorial Comments:**

E1. Please amend your detailed Financial Disclosure statement. This is published with the article. It must therefore be completed in full sentences and contain the exact wording you wish to be published.

*RESPONSE: This study was funded by the NIH National Center for Advancing Translational Sciences (NCATS UL1TR001855), the American Foundation for Suicide Prevention (ECR-0-113-23), and the Mental Research Institute.*

E1i. Please clarify all sources of financial support for your study. List the grants, grant numbers, and organizations that funded your study, including funding received from your institution. Please note that suppliers of material support, including research materials, should be recognized in the Acknowledgements section rather than in the Financial Disclosure.

*RESPONSE: This study was funded by the NIH National Center for Advancing Translational Sciences (NCATS UL1TR001855), the American Foundation for Suicide Prevention (ECR-0-113-23), and the Mental Research Institute.*

E1ii. State the initials, alongside each funding source, of each author to receive each grant. For example: "This work was supported by the National Institutes of Health (####### to AM; ###### to CJ) and the National Science Foundation (###### to AM)."
iii. State what role the funders took in the study. If the funders had no role in your study, please state: “The funders had no role in study design, data collection and analysis, decision to publish, or preparation of the manuscript.”
iv. If any authors received a salary from any of your funders, please state which authors and which funders.

*RESPONSE: This study was funded by the NIH National Center for Advancing Translational Sciences (NCATS UL1TR001855 to HO), the American Foundation for Suicide Prevention (ECR-0-113-23 to HO), and the Mental Research Institute (to HO). NCATS provided funding for data collection. The AFSP and MRI provided funding to partially cover the salary of the principal investigator (HO) at the time this manuscript was prepared. The funders had no role in the study design, data collection or analysis, decision to publish, or preparation of the manuscript.*

E2. In the online submission form, you indicated that “The questionnaire and de-identified data are available upon request. Requests should be directed to hansoh@usc.edu.”.

All PLOS journals now require all data underlying the findings described in their manuscript to be freely available to other researchers, either
1. In a public repository,
2. Within the manuscript itself, or
3. Uploaded as supplementary information.

This policy applies to all data except where public deposition would breach compliance with the protocol approved by your research ethics board. If your data cannot be made publicly available for ethical or legal reasons (e.g., public availability would compromise patient privacy), please explain your reasons by return email and your exemption request will be escalated to the editor for approval. Your exemption request will be handled independently and will not hold up the peer review process, but will need to be resolved should your manuscript be accepted for publication. One of the Editorial team will then be in touch if there are any issues.

*RESPONSE: We thank PLOS for implementing this requirement. We are committed to transparency and reproducibility and are happy to make data available to qualified researchers; however, we are unable to deposit the dataset in a fully public repository due to ethical and consent-based constraints. Although the data have been de-identified, there remains a non-trivial risk of re-identification through linkage with external datasets, particularly given the inclusion of detailed demographic information and geodata. This risk is high in mental health research and studies involving stigmatized or vulnerable populations, where breaches of confidentiality could be harmful. Additionally, participants in this study consented to their data being shared for research purposes but did not consent to unrestricted public dissemination, where data may be broadly mined or reused beyond the scope of the original study. Public release also raises concerns about potential misuse, including reinforcing stigma, enabling discriminatory profiling, or inappropriate interpretation of sensitive variables such as race or immigration-related factors. Our dataset also contains some qualitative components, and as such, full anonymization is particularly challenging since transcripts often contain contextual details (e.g., occupations, locations, personal details) that may inadvertently reveal identity if shared openly. To balance openness with participant protection, we provide data upon reasonable request through a controlled access process, which allows us to ensure appropriate use, provide necessary documentation and context, and minimize risks of misinterpretation or misuse.*

E3. Please provide separate figure files in .tif or .eps format.

For more information about figure files, please see our guidelines:

[https://journals.plos.org/digitalhealth/s/figures](https://urldefense.com/v3/__https://journals.plos.org/digitalhealth/s/figures__;!!BDUfV1Et5lrpZQ!Xbx1oZSawKwQmpM4VjuH1w8NiHCLO9-Vk1vlkqtLv-7MrlaNhihGUcsCutuieV6PJh0fIGyVGtwK86AaDl1Wt4Q$)
[https://journals.plos.org/digitalhealth/s/figures#loc-file-requirements](https://urldefense.com/v3/__https://journals.plos.org/digitalhealth/s/figures*loc-file-requirements__;Iw!!BDUfV1Et5lrpZQ!Xbx1oZSawKwQmpM4VjuH1w8NiHCLO9-Vk1vlkqtLv-7MrlaNhihGUcsCutuieV6PJh0fIGyVGtwK86Aa0RR732E$)

*RESPONSE: Thank you for this point. Separate figure files have been created in .tif format.*

**Reviewer #1:**

1. This study investigated the association between social media use and mental health issues among black emerging adults in the USA using online racism as a mediator. This study is very important since it will inform social media platforms on what features can influence online racism so that future versions may be improved. However, I have the following comments:
   a. Title: It should include United States as a context where this study took place.

*RESPONSE: Thank you for raising this point. Our manuscript is now entitled: “Social media use, online racism, and mental health among Black Emerging Adults in the United States.”*

2. Author summary - I don’t understand the first sentence, do you mean “This study suggests that Black emerging adults who frequently use specific social media platforms …”

*RESPONSE: Thank you for this note. This sentence has been edited for clarity.*

3. Introduction - Be consistent in the use of percentages, do not use both words and number .. See second paragraph - Eighty four percent of Black emerging adults report -- all along you were using numbers so maintain that. in addition, there are two full stops in this sentence -- "several times a day [10]. often for at least three hours [11]." Check the whole document for this type of mistakes.

*RESPONSE: Thank you for this note. We have revised the manuscript so that numbers have been used consistently throughout the paper, and the entire manuscript has been reviewed and revised so that there are no grammatical mistakes.*

4. Current study - Can you please make the hypothesis more prominent by listing them.

*RESPONSE: Thank you for this suggestion. The hypothesis has now been listed out.*

5. Discussion- It would be great if the design characteristics of social media platform in combating online racism could come out clearly. Remember, this is your contribution eg.
   a. 1. design characteristics that prevents users interacting with people they do not know
   b. 2. role of policies in preventing and filtering online racism
   c. 3. promote functions that enhance sense of belonging and connectedness

similarly, what is the other 3 not doing right to combat online racism?

*RESPONSE: Thank you for this note; On the bottom of page 25, we have noted how these specific social media platforms have design characteristics that “provide the opportunity to make accounts private to only specific audiences– preventing users from seeing potentially hateful content from individuals outside of close circles”. Additionally, we have clarified what particular policies help prevent online racism by emphasizing how Instagram, Facebook, and Youtube “utilize a strike system that informs users when they are in danger of violating guidelines and increases severity of punishment with higher violations” on page 26. Last, we emphasize how platforms such as Youtube create belonging through exposure to “affirming content, such as that from natural hair communities which helps to prevent exposure to online racism”, on bottom of page 26.*

6. *Minor comment- This manuscripts needs some proofreading:*

*For instance: For instance, a study with Black emerging adult users found that on average, around 85% of their Facebook friends also identified at Black [86]…*

*RESPONSE: We apologize for the errors and have carefully proofread the paper for further issues.*

Reviewer #2:

1. The introduction is a bit difficult to follow. While I appreciate the depth and rigor of the content here (especially the amount of citations), it is quite long and narratively muddled. Generally, when I read an introduction, I expect to go from the broad general context (this is established well in the paper) down to what this paper aims to achieve (this is also established well in this paper), with the sentences and paragraphs in between serving as narrative stepping stones between the two points. I feel like these stepping stones are where the introduction could use some editing (i.e., cutting, consolidating, and rewriting) to make it a smoother read. The authors should carefully consider each paragraph and what narrative purpose it is serving; some of the tangential content might be better saved for a thesis/dissertation.

*RESPONSE: Thank you for this note. The introduction has been edited for further clarity and readability, especially in areas of concern in the supporting sections.*

2. The paper doesn’t provide a justification for the focus on Black emerging adults. While this group does experience online racism, other groups do as well, and I wonder if this group is unique in some combination of the magnitude and effect of online racism or in how they use social media that warrants the specific focus, rather than a look at whether online racism acts as a mediator across multiple racial/ethnic groups. The authors should consider adding some text to this effect, either in the introduction, justifying this focus, or in the discussion/limitations section, briefly discussing the literature in this space about other groups and how these results might differ or appear similarly in those groups.

*RESPONSE: Thank you for this question. We understand the impetus to establish the importance of examining associations within a given population. Often, in writing introduction sections, this is accomplished by including statements that explicitly compare racial/ethnic groups (e.g., XYZ group has higher rates of social media use than ZYZ other groups vs. XYZ group has high rates of social media use). Racial/ethnic groups are then often examined analytically in racial/ethnic group comparative analyses. We have resisted invoking these comparisons and do not consider comparative analyses because we do not mean to justify examination of Black emerging adults as one heterogenous group only in comparison to others (e.g., "deficit" framing”), but rather support the examination of this group for its own sake. See Volpe et al. 2022 (https://doi.org/10.1111/jora.12742) and Volpe et al. 2019 (https://doi/org/10.1037/tps0000205) for discussion of these issues and our position. However, the reviewer’s points are well taken. We understand that the introduction could benefit from acknowledgement that other groups experience online racism as well. Therefore, we have added the following information to the discussion: “Although members of other racial/ethnic groups also experience online racism, in the current study we focus on the experiences of Black emerging adults as one heterogeneous marginalized group with high mental health risk and high rates of social media use.” We have also added the following information to the limitations and future directions section, “Finally, other investigations may consider these issues with other racial/ethnic groups to understand if results might differ or appear similarly in those groups”.*

3. Page 8: “outcomes in offline settings [e.g., 51-52].” Are these citations? Other citations are links, whereas these were not, so just wanted to note that these might warrant double-checking.

*RESPONSE: Thank you. These hyperlinks have been double-checked and fixed.*

4. In Table 1, what is the unit for social media use? Is this mean and standard deviation of hours per day?

*RESPONSE: Thank you for this question. Responses were made on a Likert-type scale with the following options: 0 (Not at all), 1 (Less than a few times a day), 2 (A few times a day), 3 (Many times a day), and 4 (Almost constantly). This information has now been included in the note of the Table, so that readers can more readily interpret the numbers presented with regard to the response scale anchors (e.g., a mean score of 3.12 suggests “many times a day” frequency).*

5. Page 15: “Therefore, in the current study, we aim to understand if negative experiences on social media (e.g., online racism) are one potential explanation.” Shouldn’t this be i.e.? If it’s e.g., what other negative experiences are you looking at here?

*RESPONSE: Thank you for pointing this out; we have changed it to “i.e.,”.*

6. As noted by the submission guidelines, please also include line numbers in the revised manuscript to make it easier for reviewers to refer to specific parts of the manuscript.

*RESPONSE: Thank you for this comment. We have added line numbers in the revised manuscript.*

Reviewer #3:

1. On pages 6 and 25, please swap "increased odds" for "was associated with increased odds" to ensure the language matches the cross-sectional nature of your study design.

*RESPONSE: Thank you for this note. Both examples included the use of “as associated with increased odds”.*

2. It would be helpful if you described the actual content or logic of the two attention check questions on page 15 so that future researchers can replicate your data quality standards.

*RESPONSE: The attention check questions were as follows:*

*The following question is to verify you are a real person. Which of the following is a vegetable? Hamburger, Broccoli, Pizza, Milk.*

*Please select ‘strongly agree’ to show that you are paying attention. Strongly disagree, disagree, somewhat agree, strongly agree.*

*Explanations for attention checks were added on page 12: “participants were asked to verify which item was a vegetable in a list of food items, and select ‘strongly agree’ on a Likert scale to show they were paying attention.”*

3. Please add a brief note on page 19 explaining why education level or employment status weren't included as controls, as these typically influence mental health in this demographic.

*RESPONSE: Thank you for this important note. We did measure education level and employment status as potential covariates for inclusion. However, when we tested them in the current sample, they were not statistically significantly associated with depression (education 𝜒^2^(7) = 5.32, p = .621. ; employment 𝜒^2^(1) = .10, p = .755) or anxiety (education 𝜒^2^(7) = 5.66, p = .580; employment 𝜒^2^(1) = .38, p = .539). We have now included this information as a footnote in the sociodemographic covariates section for interested readers, writing: “*We also did consider education level and employment status as potential sociodemographic covariates. However, when we tested them in the current sample, they were not statistically significantly associated with depression (education 𝜒2(7) = 5.32, *p* = .621. ; employment 𝜒2(1) = .10, *p* = .755) or anxiety (education 𝜒2(7) = 5.66, *p* = .580; employment 𝜒2(1) = .38, *p* = .539). Therefore, we did not retain them in our analytic models.”

4. In Figure 1 on page 10, I suggest adding the standardized coefficients for the significant paths found for Twitter, Reddit, and TikTok to make the core findings clear at a glance.

*RESPONSE: Thank you for this note. If we understand your comment correctly, you may be looking for betas (standardized linear regression coefficients) for the paths between social media platforms and the outcome of mental health variables in Table 1 (as there is no figure with findings of the paths). As the outcome of this part of the mediation model is dichotomous (mental health outcomes as the outcome social media use as the predictor) we provide the Odds Ratio in this column for these paths in the first part of the Table. However we name the column heading “OR (95% CI or* β *(95% CI)” because - for the paths from social media platforms to online racism outcome (i.e., the paths to the mediator) these are indeed standardized betas (*β*) because the online racism intermediary outcome is continuous.*

5. On page 31, please provide a more specific example of a "platform-specific coping strategy" that a clinician could actually suggest, going beyond just "turning off notifications"

*RESPONSE: We appreciate this suggestion to provide more tangible strategies. In response we added “prioritizing content created by trusted creators” and “blocking specific terms and hashtags.”*

6. On page 30, please explicitly state in your limitations that using a non-probability online sample may have selected for individuals who are more digitally active and thus more likely to encounter online racism.

*RESPONSE: Thank you for this note; we have added this to our limitations.*

7. In Table 2 on page 22, the Anxiety column is quite cramped; please add clear spacing between the 95% CI brackets and the p-values to improve readability.

*RESPONSE:*
