# Supplementary material for: Social media use, online racism, and mental health among Black Emerging Adults in the United States
Source: PLOS Digit Health. 2026 Aug 13;5(8):e0001243. doi: 10.1371/journal.pdig.0001243 (PMC13472414; doi:10.1371/journal.pdig.0001243)
Supplement: Peer review history

# Peer review: Decision Letter 0

**DOI:** 10.1371/journal.pdig.0001243.r001

**Authors:** Po-Chih Kuo, Girum Tareke Zewude

8 Apr 2026

PDIG-D-25-01279

Social media use, online racism, and mental health among Black Emerging Adults

PLOS Digital Health

Dear Dr. Oh,

Thank you for submitting your manuscript to PLOS Digital Health. After careful consideration, we feel that it has merit but does not fully meet PLOS Digital Health's publication criteria as it currently stands. Therefore, we invite you to submit a revised version of the manuscript that addresses the points raised during the review process.

Please submit your revised manuscript by Jun 07 2026 11:59PM. If you will need more time than this to complete your revisions, please reply to this message or contact the journal office at digitalhealth@plos.org. When you're ready to submit your revision, log on to https://www.editorialmanager.com/pdig/ and select the 'Submissions Needing Revision' folder to locate your manuscript file.

Please include the following items when submitting your revised manuscript:

* A letter that responds to each point raised by the editor and reviewer(s). You should upload this letter as a separate file labeled 'Response to Reviewers'. This file does not need to include responses to any formatting updates and technical items listed in the 'Journal Requirements' section below.

* A marked-up copy of your manuscript that highlights changes made to the original version. You should upload this as a separate file labeled 'Revised Manuscript with Track Changes'.

* An unmarked version of your revised paper without tracked changes. You should upload this as a separate file labeled 'Manuscript'.

If you would like to make changes to your financial disclosure, competing interests statement, or data availability statement, please make these updates within the submission form at the time of resubmission. Guidelines for resubmitting your figure files are available below the reviewer comments at the end of this letter.

We look forward to receiving your revised manuscript.

Kind regards,

Girum Tareke Zewude

Academic Editor

PLOS Digital Health

Hyunjung Kwak

Section Editor

PLOS Digital Health

Leo Anthony Celi

Editor-in-Chief

PLOS Digital Health

orcid.org/0000-0001-6712-6626

**Journal Requirements:**

1.  Please amend your detailed Financial Disclosure statement. This is published with the article. It must therefore be completed in full sentences and contain the exact wording you wish to be published.

i. Please clarify all sources of financial support for your study. List the grants, grant numbers, and organizations that funded your study, including funding received from your institution. Please note that suppliers of material support, including research materials, should be recognized in the Acknowledgements section rather than in the Financial Disclosure.

ii. State the initials, alongside each funding source, of each author to receive each grant. For example: "This work was supported by the National Institutes of Health (####### to AM; ###### to CJ) and the National Science Foundation (###### to AM)."

iii. State what role the funders took in the study. If the funders had no role in your study, please state: “The funders had no role in study design, data collection and analysis, decision to publish, or preparation of the manuscript.”

iv. If any authors received a salary from any of your funders, please state which authors and which funders.

2. In the online submission form, you indicated that “The questionnaire and de-identified data are available upon request. Requests should be directed to hansoh@usc.edu.”.

All PLOS journals now require all data underlying the findings described in their manuscript to be freely available to other researchers, either

1. In a public repository,

2. Within the manuscript itself, or

3. Uploaded as supplementary information.

This policy applies to all data except where public deposition would breach compliance with the protocol approved by your research ethics board. If your data cannot be made publicly available for ethical or legal reasons (e.g., public availability would compromise patient privacy), please explain your reasons by return email and your exemption request will be escalated to the editor for approval. Your exemption request will be handled independently and will not hold up the peer review process, but will need to be resolved should your manuscript be accepted for publication. One of the Editorial team will then be in touch if there are any issues.

3. Please provide separate figure files in .tif or .eps format.

For more information about figure files please see our guidelines:

https://journals.plos.org/digitalhealth/s/figures

https://journals.plos.org/digitalhealth/s/figures#loc-file-requirements

If the reviewer comments include a recommendation to cite specific previously published works, please review and evaluate these publications to determine whether they are relevant and should be cited. There is no requirement to cite these works unless the editor has indicated otherwise.

**Additional Editor Comments (if provided):**

[Note: HTML markup is below. Please do not edit.]

**Reviewers' Comments:**

Reviewer's Responses to Questions

**Comments to the Author**

1. Does this manuscript meet PLOS Digital Health’s publication criteria? Is the manuscript technically sound, and do the data support the conclusions? The manuscript must describe methodologically and ethically rigorous research with conclusions that are appropriately drawn based on the data presented.

Reviewer #1: Yes

Reviewer #2: Yes

Reviewer #3: Partly

**********

2. Has the statistical analysis been performed appropriately and rigorously?

Reviewer #1: Yes

Reviewer #2: Yes

Reviewer #3: Yes

**********

3. Have the authors made all data underlying the findings in their manuscript fully available (please refer to the Data Availability Statement at the start of the manuscript PDF file)?

The PLOS Data policy requires authors to make all data underlying the findings described in their manuscript fully available without restriction, with rare exception. The data should be provided as part of the manuscript or its supporting information, or deposited to a public repository. For example, in addition to summary statistics, the data points behind means, medians and variance measures should be available. If there are restrictions on publicly sharing data—e.g. participant privacy or use of data from a third party—those must be specified.

Reviewer #1: No

Reviewer #2: Yes

Reviewer #3: Yes

**********

4. Is the manuscript presented in an intelligible fashion and written in standard English?

PLOS Digital Health does not copyedit accepted manuscripts, so the language in submitted articles must be clear, correct, and unambiguous. Any typographical or grammatical errors should be corrected at revision, so please note any specific errors here.

Reviewer #1: Yes

Reviewer #2: Yes

Reviewer #3: Yes

**********

5. Review Comments to the Author

Please use the space provided to explain your answers to the questions above. You may also include additional comments for the author, including concerns about dual publication, research ethics, or publication ethics. (Please upload your review as an attachment if it exceeds 20,000 characters)

**Reviewer #1:**  This study investigated the association between social media use and mental health issues among black emerging adults in the USA using online racism as a mediator. This study is very important since it will inform social media platforms on what features can influence online racism so that future versions may be improved. However, I have the following comments:

1. Title: It should include United States as a context where this study took place.

2. Author summary

I don’t understand the first sentence, do you mean “This study suggests that Black emerging adults who frequently use specific social media platforms …”

3. Introduction

Be consistent in the use of percentages, do not use both words and number .. See second paragraph - Eighty four percent of Black emerging adults report -- all along you were using numbers so maintain that. in addition, there are two full stops in this sentence -- "several times a day [10]. often for at least three hours [11]." Check the whole document for this type of mistakes.

Current study

Can you please make the hypothesis more prominent by listing them.

4. Discussion

It would be great if the design characteristics of social media platform in combating online racisms could come out clearly. Remember, this is your contribution eg

1. design characteristics that prevents users interacting with people they do not know

2. role of policies in preventing and filtering online racism

3. promote functions that enhance sense of belonging and connectedness

similarly, what is the other 3 not doing right to combat online racism?

This will help the reader to get the takeaways of your study more easily.

Minor comment

This manuscripts needs some proofreading:

For instance:

“For instance, a study with Black emerging adult users found that on average, around

85% of their Facebook friends also identified at Black [86]…

**Reviewer #2:**  This work looks at whether online racism acts as a mediator of the association between social media use and depression and anxiety symptoms. Overall, it is well written, rigorous, and should be of interest to the PLOS Digital Health audience. I have a few comments that should be addressable with a minor revision.

The introduction is a bit difficult to follow. While I appreciate the depth and rigor of the content here (especially the amount of citations), it is quite long and narratively muddled. Generally, when I read an introduction, I expect to go from the broad general context (this is established well in the paper) down to what this paper aims to achieve (this is also established well in this paper), with the sentences and paragraphs in between serving as narrative stepping stones between the two points. I feel like these stepping stones are where the introduction could use some editing (i.e., cutting, consolidating, and rewriting) to make it a smoother read. The authors should carefully consider each paragraph and what narrative purpose it is serving; some of the tangential content might be better saved for a thesis/dissertation.

The paper doesn’t provide a justification for the focus on Black emerging adults. While this group does experience online racism, other groups do as well, and I wonder if this group is unique in some combination of the magnitude and effect of online racism or in how they use social media that warrants the specific focus, rather than a look at whether online racism acts as a mediator across multiple racial/ethnic groups. The authors should consider adding some text to this effect, either in the introduction, justifying this focus, or in the discussion/limitations section, briefly discussing the literature in this space about other groups and how these results might differ or appear similarly in those groups.

Minor Points

Page 8: “outcomes in offline settings [e.g., 51-52].” Are these citations? Other citations are links, whereas these were not, so just wanted to note that these might warrant double-checking.

In Table 1, what is the unit for social media use? Is this mean and standard deviation of hours per day?

Page 15: “Therefore, in the current study, we aim to understand if negative experiences on social media (e.g., online racism) are one potential explanation” Shouldn’t this be i.e.? If it’s e.g., what other negative experiences are you looking at here?

As noted by the submission guidelines, please also include line numbers in the revised manuscript to make it easier for reviewers to refer to specific parts of the manuscript.

Reviewer #3: This research tests whether online racism explains why frequent use of certain social media platforms correlates with depression and anxiety in Black emerging adults.

You’ve addressed a major gap by moving away from generic "screen time" to examine how specific platform environments like Reddit’s anonymity versus Instagram’s social networks impact mental health via racial stressors.

On pages 6 and 25, please swap "increased odds" for "was associated with increased odds" to ensure the language matches the cross-sectional nature of your study design.

It would be helpful if you described the actual content or logic of the two attention check questions on page 15 so that future researchers can replicate your data quality standards.

Please add a brief note on page 19 explaining why education level or employment status weren't included as controls, as these typically influence mental health in this demographic.

In Figure 1 on page 10, I suggest adding the standardized coefficients for the significant paths found for Twitter, Reddit, and TikTok to make the core findings clear at a glance.

On page 31, please provide a more specific example of a "platform-specific coping strategy" that a clinician could actually suggest, going beyond just "turning off notifications"

On page 30, please explicitly state in your limitations that using a non-probability online sample may have selected for individuals who are more digitally active and thus more likely to encounter online racism.

In Table 2 on page 22, the Anxiety column is quite cramped; please add clear spacing between the 95% CI brackets and the p-values to improve readability.

Over all its a good manuscript . All the best

**********

6. PLOS authors have the option to publish the peer review history of their article (what does this mean?). If published, this will include your full peer review and any attached files.

**Do you want your identity to be public for this peer review?**  If you choose “no”, your identity will remain anonymous but your review may still be made public.

For information about this choice, including consent withdrawal, please see our Privacy Policy.

Reviewer #1: **Yes:** Priscilla Maliwichi

Reviewer #2: No

Reviewer #3: **Yes:** Bala Nimmana

**********

[NOTE: If reviewer comments were submitted as an attachment file, they will be attached to this email and accessible via the submission site. Please log into your account, locate the manuscript record, and check for the action link "View Attachments". If this link does not appear, there are no attachment files.]

**Figure resubmission:**

While revising your submission, we strongly recommend that you use PLOS’s NAAS tool (https://ngplosjournals.pagemajik.ai/artanalysis) to test your figure files. NAAS can convert your figure files to the TIFF file type and meet basic requirements (such as print size, resolution), or provide you with a report on issues that do not meet our requirements and that NAAS cannot fix.

After uploading your figures to PLOS’s NAAS tool - https://ngplosjournals.pagemajik.ai/artanalysis, NAAS will process the files provided and display the results in the "Uploaded Files" section of the page as the processing is complete. If the uploaded figures meet our requirements (or NAAS is able to fix the files to meet our requirements), the figure will be marked as "fixed" above. If NAAS is unable to fix the files, a red "failed" label will appear above. When NAAS has confirmed that the figure files meet our requirements, please download the file via the download option, and include these NAAS processed figure files when submitting your revised manuscript.

**Reproducibility:**

To enhance the reproducibility of your results, we recommend that authors of applicable studies deposit laboratory protocols in protocols.io, where a protocol can be assigned its own identifier (DOI) such that it can be cited independently in the future. Additionally, PLOS ONE offers an option to publish peer-reviewed clinical study protocols. Read more information on sharing protocols at https://plos.org/protocols?utm_medium=editorial-email&utm_source=authorletters&utm_campaign=protocols

# Author comment: Author response to Decision Letter 1

**DOI:** 10.1371/journal.pdig.0001243.r002

7 Jun 2026

Attachment Submitted filename: Response to Reviewers.docx

# Peer review: Decision Letter 1

**DOI:** 10.1371/journal.pdig.0001243.r003

**Authors:** Po-Chih Kuo, Girum Tareke Zewude

29 Jun 2026

Social media use, online racism, and mental health among Black Emerging Adults in the United States

PDIG-D-25-01279R1

Dear Dr. Oh,

We are pleased to inform you that your manuscript 'Social media use, online racism, and mental health among Black Emerging Adults in the United States' has been provisionally accepted for publication in PLOS Digital Health.

Before your manuscript can be formally accepted you will need to complete some formatting changes, which you will receive in a follow-up email from a member of our team.

Please note that your manuscript will not be scheduled for publication until you have made the required changes, so a swift response is appreciated.

IMPORTANT: The editorial review process is now complete. PLOS will only permit corrections to spelling, formatting or significant scientific errors from this point onwards. Requests for major changes, or any which affect the scientific understanding of your work, will cause delays to the publication date of your manuscript.

If your institution or institutions have a press office, please notify them about your upcoming paper to help maximize its impact. If they'll be preparing press materials, please inform our press team as soon as possible -- no later than 48 hours after receiving the formal acceptance. Your manuscript will remain under strict press embargo until 2 pm Eastern Time on the date of publication. For more information, please contact digitalhealth@plos.org.

Thank you again for supporting Open Access publishing; we are looking forward to publishing your work in PLOS Digital Health.

Best regards,

Girum Tareke Zewude, PhD

Section Editor

PLOS Digital Health

***********************************************************

**Additional Editor Comments (if provided):**

**Reviewer Comments (if any, and for reference):**

Reviewer's Responses to Questions

**Comments to the Author**

1. If the authors have adequately addressed your comments raised in a previous round of review and you feel that this manuscript is now acceptable for publication, you may indicate that here to bypass the “Comments to the Author” section, enter your conflict of interest statement in the “Confidential to Editor” section, and submit your "Accept" recommendation.

Reviewer #2: All comments have been addressed

Reviewer #3: All comments have been addressed

Reviewer #4: (No Response)

**********

2. Does this manuscript meet PLOS Digital Health’s publication criteria? Is the manuscript technically sound, and do the data support the conclusions? The manuscript must describe methodologically and ethically rigorous research with conclusions that are appropriately drawn based on the data presented.

Reviewer #2: Yes

Reviewer #3: Yes

Reviewer #4: Yes

**********

3. Has the statistical analysis been performed appropriately and rigorously?

Reviewer #2: Yes

Reviewer #3: Yes

Reviewer #4: Yes

**********

4. Have the authors made all data underlying the findings in their manuscript fully available (please refer to the Data Availability Statement at the start of the manuscript PDF file)?

The PLOS Data policy requires authors to make all data underlying the findings described in their manuscript fully available without restriction, with rare exception. The data should be provided as part of the manuscript or its supporting information, or deposited to a public repository. For example, in addition to summary statistics, the data points behind means, medians and variance measures should be available. If there are restrictions on publicly sharing data—e.g. participant privacy or use of data from a third party—those must be specified.

Reviewer #2: Yes

Reviewer #3: Yes

Reviewer #4: No

**********

5. Is the manuscript presented in an intelligible fashion and written in standard English?

PLOS Digital Health does not copyedit accepted manuscripts, so the language in submitted articles must be clear, correct, and unambiguous. Any typographical or grammatical errors should be corrected at revision, so please note any specific errors here.

Reviewer #2: Yes

Reviewer #3: Yes

Reviewer #4: Yes

**********

6. Review Comments to the Author

Please use the space provided to explain your answers to the questions above. You may also include additional comments for the author, including concerns about dual publication, research ethics, or publication ethics. (Please upload your review as an attachment if it exceeds 20,000 characters)

**Reviewer #2** : I am satisfied with the revisions and responses the reviewers have provided. Two minor suggestions related to responses to reviewer 1: I would suggest the authors remove the number from before their hypothesis, a list of one item seems a bit silly to me. The reviewers can simply style this like a block quote to ensure the hypothesis still stands out. Also I recognize the reviewer asked the authors to be consistent in how they refer to percentages, but the convention is to spell out numbers at the start of sentences and this inconsistency with writing them as numbers elsewhere is normal. Regardless, these are nitpicks and I leave it up to the authors as to whether they wish to change these things.

**Reviewer #3:**  The authors have done a very good job addressing the previous round of feedback, particularly by restructuring the introduction to improve narrative flow and comprehensively updating their financial disclosures.

The inclusion of "in the United States" in the title successfully clarifies the geographic boundaries and cultural context of the study population.

The introduction reads much more smoothly now.

The added discussion text regarding the specific content moderation policies (such as Meta's strike system versus X's warning labels) provides excellent depth regarding design characteristics.

**Reviewer #4:**  Dear authors,

The manuscript entitled "Social media use, online racism, and mental health among Black Emerging Adults in

the United States" presents interesting data regarding Black American's usage of social media and online racism. I recommend minor revisions. Please see my recommendations below.

- Similar to the previous reviewer, I would recommend referring to the study population as "Black Americans" rather than just "Black" - as this is an international journal I believe this would provide clarity.

- In the materials and methods section, you mention that you validated the participants credentials (Lines 267-269). Can you clarify what credentials were reviewed and why these methods were used?

- Table 1 seems to belong in the results rather than the methods section

- I would suggest adding units or a note to Table 1 to clarify that the results reported in the "Social Media Use" section are Likert scale scores. Initially I thought these might be percentages

**********

7. PLOS authors have the option to publish the peer review history of their article (what does this mean?). If published, this will include your full peer review and any attached files.

**Do you want your identity to be public for this peer review?**  If you choose “no”, your identity will remain anonymous but your review may still be made public.

For information about this choice, including consent withdrawal, please see our Privacy Policy.

Reviewer #2: No

Reviewer #3: **Yes:** Bala Nimmana

Reviewer #4: No

**********
